# Quality of life of patients with first-time AMI: a descriptive study

**DOI:** 10.1186/s12955-018-0860-8

**Published:** 2018-02-13

**Authors:** Mandreker Bahall, Katija Khan

**Affiliations:** Department of Clinical Medical Sciences, University of the West Indies, St. Augustine, Trinidad Trinidad and Tobago

**Keywords:** Patients with AMI, QOL, Clinical outcome, Time series

## Abstract

**Background:**

Outcomes following acute myocardial infarction (AMI) may result in death, increased morbidity, and change in quality of life (QOL). This study explores health-related QOL of first-time patients following AMI.

**Methods:**

This cross-sectional study used a sample of patients with first-time AMI experienced between April 2011 and March 2015 at a tertiary health institution. Recruited patients belonged to different post-AMI periods: 2–10 weeks, 5–22 months, and > 22 months to 4 years post AMI. Inclusion criteria were not confused and communicating freely. Exclusion criteria were non-contactable, refusing to participate, and deceased. One-on-one interviews were conducted using the validated and pre-tested Quality of Life after Myocardial Infarction (QLMI) questionnaire. QOL of patients after AMI was evaluated at each period. Descriptive, Mann–Whitney U, Kruskal–Wallis, and regression analyses were conducted using SPSS version 24.

**Results:**

A total of 534 participant interviews (overall response rate 65.4%) were conducted. Interviewees were predominantly male (67%), aged 51–65 years (45%), Indo-Trinidadian (81.2%), NSTEMI (64.4%), and hypertensive (72.4%). Overall QOL improved over time and in all domains: Emotional, Physical, and Social. Lower QOL was found among women, patients with NSTEMI, and diabetics in all domains; in patients with hypertension and renal disease in the Physical and Social domains only; and in patients with ischaemic heart disease (IHD) in the Physical domain only. Self-reported stress and lack of exercise were associated with lower QOL while drinking alcohol and eating out were related to better QOL. Hypercholesterolemia, smoking, and ethnicity showed no association with QOL. Declining QOL in the Physical domain with age was also found. The leading components of QOL were self-confidence and social exclusion (early post AMI), lack of self-confidence (intermediate post AMI), and tearfulness (late post AMI).

**Conclusions:**

QOL in AMI survivors improves over time. Female gender, NSTEMI, diabetes, hypertension, renal disease, stress, and lack of exercise were associated with lower QOL while hypercholesterolemia, smoking, and ethnicity showed no association with QOL. Cardiac rehabilitation and psychological support may enhance earlier increased QOL among survivors, particularly among vulnerable groups.

## Background

Major outcome measures of acute myocardial infarction (AMI) include physical, social, and psychological domains [1]. Quality of life (QOL) is dependent not only on health provider factors such as access to medical care [2], cardiovascular risk factors [3], and severity of AMI [3], but also on demographic and psychosocial factors such as age [4], sex [5], educational level [6], income [6], and family and social support [7]. Following AMI, many patients are left traumatized emotionally and physically [8]. Such feelings and an initial reaction of anxiety are aggravated by the perception that a heart attack could lead to imminent death, a belief that affects patients, relatives, and the community even after receiving adequate medical treatment [9]. Reports of family problems and alterations in family life [10], physical activity phobias [11], depression [12], and stress [12] are common, leading to further decline in physical activity and household economic burden [13]. In contrast, improved clinical care leads to a decrease in mortality [14] [15] and improvement in QOL and clinical outcomes [15], which can further increase life expectancy and decrease morbidity [16]. In this regard, it is not enough to add years to a person’s life; life must also be added to those years [17].

AMI incidence in South Trinidad is 90.6 per 100,000 population with ethnic variation: 55.3 Afro-Trinidadians and 106.6 Indo-Trinidadians per 100,000 population [18]. CAD ranks among the 10 most common chronic diseases globally and is the number one cause of death [19] [20] in Trinidad and Tobago. Every effort must therefore be given to improve the quality of life of patients with CAD. As stated by the World Health Organization (WHO), “quality of life is defined as individuals’ perceptions of their position in life in the context of the culture and value systems in which they live and in relation to their goals, expectations, standards, and concerns” [21]. It incorporates many different aspects of a person’s life including: physical, psychological, level of independence, social relations, environmental, and spiritual/religion/personal beliefs [22]. Health-related QOL of life is defined as a “multi-dimensional concept that includes domains related to physical, mental, emotional, and social functioning”. [23]

Although health-related QOL is an important patient outcome [24], it has not been routinely monitored and measured in local settings. Measuring a patient’s QOL is of immense value to determine care and to identify appropriate interventions to optimize care. Over the last few decades, numerous QOL tools have been developed to measure patients’ QOL. The Quality of Life after Myocardial Infarction (QLMI) questionnaire, however, was developed specifically for post-AMI patients [25], has shown good reliability [26], and was chosen for use in this study. The purpose of this descriptive cross-sectional study was to determine health-related QOL and related factors in patients after AMI at different time periods.

## Methods

### Setting

This study was conducted in a public tertiary health care institution (San Fernando General Hospital) in Trinidad and Tobago (population of 1.3 million people). The institution provides free services covering a variety of investigations, treatment, and follow-up care. Surgical interventions such as angioplasty, open heart surgery, and other mechanical device implants are also provided freely through the Ministry of Health’s Cardiac Assistance Programme. However, lifestyle interventions such as exercise, diet, relaxation therapy, cardiac rehabilitation, and psychotherapy, which have all been recognized to improve QOL [27], have not been emphasized.

### Participants

**Fig. 1:**
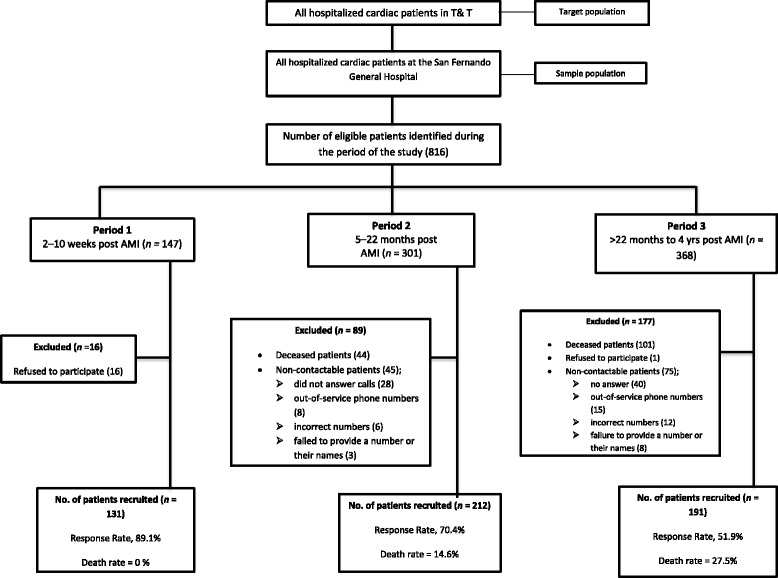
Participants selection. AMI = acute myocardial infarction

Patients for this study were drawn from the Myocardial Infarction in South Trinidad (MIST) database of patients with first-time AMI admitted between 2011 and 2015. AMI was defined as the experience of ischemic symptoms with or without significant ECG changes, and a change in troponin level [28]. While QOL status of post-AMI patients in Trinidad has not been investigated, relevant studies have been conducted in other countries using a range of different times post AMI: for example, 1 month (*N* = 160) [29], 2 weeks to 3 months [30], 3 months (*N* = 132) [31], 1 to 12 months (*N* = 4340) [32], 6 to 12 months (*N* = 587) [33], 4 years (*N* = 900) [34], and an unknown time period (*N* = 2950) [35]. These studies reveal wide variation in time and sample sizes. With no precedent or existing guidelines for specific time periods, this study utilised three time periods to correspond roughly to shortly after, within 2 years, and after 2 years. Specifically, these were the following: 2 to 10 weeks post AMI (Period 1), 5 to 22 months post AMI (Period 2), and over 22 weeks to 4 years post AMI (Period 3). Inclusion criteria were first-time AMI patients and not confused (ability to understand, think clearly, and make meaningful understandable statements). Exclusion criteria included those who felt physically challenged and unable to attend an interview, unwilling, death, and non-contactable patients (after at least 3 attempts by phone). A breakdown of the patients selected (included and excluded) in each period is depicted in Fig. 1.

### Instruments

Patients with AMI were asked to describe their QOL status using the QLMI questionnaire, which was pilot tested with 10 patients to ensure comfort with the wording and understanding of the questions. The QLMI is a 7-point, frequency-based Likert scale comprising 27 questions covering emotional, physical, and social domains. Lower scores correspond to a lower quality of life. Other data extracted from the MIST database included socio-demographics: age, gender, ethnicity, income, educational attainment, job status, type of AMI (ST-segment elevation myocardial infarction, STEMI; or non–ST-segment elevation myocardial infarction, NSTEMI), and medical history.

Interviews were conducted either at the hospital (with patients who agreed to return to the hospital for the interview), the patient’s home, or a location convenient for the patient. Some interviews were conducted on the phone, when the patient preferred this method or could not physically attend the interview. Data extracted were entered in a secured computer database that could be accessed only by the researchers, statistician, and research assistants.

### Analysis

Data analysis was conducted using SPSS version 24. Given the unequal group sizes and lack of homogeneity of variance among groups, non-parametric tests were used. Mann-Whitney U and Kruskal–Wallis tests were used to compare group differences in scores on QOL and to identify associations with demographic, clinical, and lifestyle factors. Analyses were confined to single factors at a time as combined analysis of multiple variables together was deemed inappropriate because of small sample size and unreliability of subgroup analysis. Linear regression analyses were used to compare test indicators within the different domains of the QLMI, and to identify specific dimensions of Quality of Life most endorsed in the different time periods.

## Results

The response rate was 65.4% (*n* = 816). Mortality rate was highest (27.4%) in the period from > 22 months to 4 years, followed by 14.6% in the period from 5 to 22 months. Patient interviewees were predominantly male (*n* = 342, 67%), aged 51–65 years (*n* = 126, 45%), Indo-Trinidadian (*n* = 417, 81.2%), NSTEMI (*n* = 255, 64.4%), and hypertensive (*n* = 329, 72.4%). The mean age of the sample was 60.57 years (SD = 12.56). Participants were further categorised into ages 29–50, 51–65, and > 65 years. There were no significant differences by gender or ethnicity among the age categories (see Table 1).

**Table 1:** Demographic, clinical, and lifestyle characteristics of the sample

	2–10 wks post MI		5–22 mths post MI		> 22 months–4 yrs. post MI		Total
Description	n	%	n	%	n	%	n
Gender							
Male	63	48.1	151	71.2	128	67	342
Female	68	51.9	61	28.8	63	33	192
Total	131	100	212	100	191	100	534
Age group (years)							
29–50	25	19.1	42	19.8	36	18.8	147
51–65	60	45.8	105	49.5	86	45	126
> 65	46	35.1	65	30.7	69	36.1	137
Total	131	100	212	100	191	100	534
Ethnicity							
Indo-Trinidadian	89	67.9	173	81.6	155	81.2	417
Afro-Trinidadian	22	16.8	27	12.7	31	16.2	80
Mixed-Trinidadian	20	15.3	12	5.7	5	2.6	37
Total	131	100	212	100	191	100	534
AMI							
STEMI	17	13.0	124	58.5	68	35.6	209
NSTEMI	44	33.6	88	41.5	123	64.4	255
Missing	70	46.6	0	0	0	0	70
Total	131	100	212	100	191	100	534
Clinical factors							
Diabetes	57	43.5	135	63.7	106	58.6	279
Hypertension	65	49.6	151	71.2	131	72.4	329
Hypercholesterolemia	49	37.4	29	13.7	53	29.3	105
Ischemic heart disease	35	26.7	101	47.6	79	44.4	211 6-
Kidney problems	11	8.4	35	16.5	19	9.9	65
Lifestyle factors							
Smoking	28	21.4	25	11.8	15	7.9	68
Alcohol	48	36.6	78	36.8	79	41.4	205
Stress	30	22.9	60	28.3	63	33.3	153
Eating out (≥1/week)	7	5.3	32	15.1	48	25.1	86
Exercise (≥20mins/wk)	30	22.9	68	32.1	77	30.3	172

### QOL and post AMI time periods

**Fig. 2:**
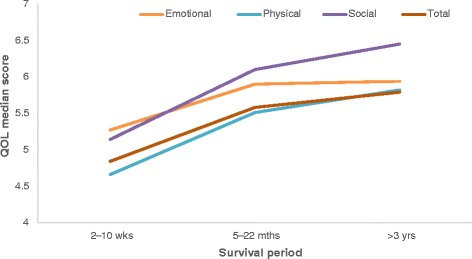
Quality of life over time in post-AMI patients

Using a Kruskal-Wallis H test, statistically significant differences were observed in all QOL domains in each period: Emotional (χ2(2) = 48.79, *p* < .001, *n* = 486), Physical (χ2(2) = 55.68, p < .001, *n* = 482), Social (χ2(2) = 85.51, p < .001, *n* = 485), and overall/total QOL (χ2(4) = 55.68, p < .001, n = 482). QOL scores were highest in the > 22 months to 4 years survivor group followed by the 5–22 months survivor group, with the early post-AMI group (2–10 weeks) having the lowest QOL scores. This trend was observed across all the QOL domains (Fig. 2).

### QOL and demographic factors

**Fig. 3:**
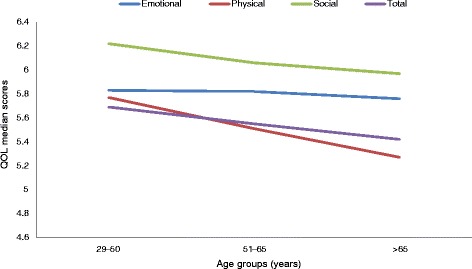
Median scores on QOL domains by age

Self-reported QOL was higher for males (*n* = 318) than females (*n* = 198) in all domains: Emotional (U = 22,319.5, *p* = 0.003), Physical (U = 21,914.0, p = 0.003), Social (U = 23,765.5, *p* = 0.048), and overall (U = 21,397.5, *p* = 0.004). Although Afro-Trinidadians had higher median scores than Indo-Trinidadians and the mixed group, there were no statistically significant differences in QOL domains by ethnicity: Emotional (χ2(2) =3.79, *p* = .150, *n* = 486), Physical (χ2(2) = 1.55, *p* = .460, *n* = 482), Social (χ2(2) = 2.56, *p* = .279, *n* = 485), and overall/total QOL (χ2(2) = 2.23, *p* = .329, *n* = 476). With respect to age groups, significant differences were only found for the Physical domain (χ2(2) = 13.686, *p* = 0.001) and Total scores (χ2(2) = 7.652, *p* = 0.027). The trend in scores show the lowest QOL scores for the oldest age group (> 65 years) when compared to the younger age groups (Fig. 3).

### QOL and clinical factors

Clinical factors exerted a varying effect on the different aspects of QOL. Between the two types of AMI (STEMI and NSTEMI), there were significant group differences for Emotional (U = 19,123.500, *p* = 0.002), Physical (U = 19,304.000, *p* = 0.005), Social (U = 20,175.000, *p* = 0.021), and Total QOL (U = 18,557, *p* = 0.003), such that patients with STEMI had significantly better QOL scores than patients with NSTEMI. Patients with diabetes (*n* = 275) had significantly lower QOL scores than patients without diabetes (*n* = 170) in all domains: Emotional (U = 20,648, *p* = 0.038), Physical (U = 18,659, *p* = 0.001), Social (U = 17,782, *p* < 0.001) and total QOL (U = 18,655, *p* = 0.002). Patients with hypertension had significantly lower scores in the Physical domain (U = 16,126, *p* = 0.003), Social domain (U = 17,104, *p* = 0.018), and Total QOL (U = 16,459,*p* = 0.016), but not the Emotional domain. Similarly, patients with kidney problems had significantly lower scores than patients with no kidney problems for the Physical domain (U = 8323, p = 0.003), Social domain (U = 8751, p = 0.016), and Total QOL (U = 8181, *p* = 0.005), but not the Emotional domain. Patients with IHD differed significantly only for the Physical domain in which they had significantly lower scores (U = 20,187, *p* = 0.021). No significant differences were observed for QOL between patients with or without hypercholesterolemia.

### QOL and lifestyle factors

A Mann-Whitney U analysis revealed eating out once a week or more was associated with significantly higher QOL in the Physical (U = 11,640, *p* = 0.001), Social (U = 11,446, *p* < 0.001), and Total QOL (U = 12,143, *p* = 0.004) domains, but not the Emotional domain. Patients with a self-reported stressful life had significantly lower median scores in all domains of QOL: Emotional (U = 15,094, *p* < 0.001), Physical (U = 17,421, p < 0.001), Social (U = 19,612, *p* = 0.015) and overall/total scores (U = 16,696, p < 0.001). Patients who exercised had significantly higher QOL scores in all domains compared to patients who did not exercise: Emotional (U = 18,533, p < 0.001), Physical (U = 16,921, p < 0.001), Social (U = 18,282, p < 0.001), and Total domains (U = 16,732, p < 0.001).

QOL scores were similar across all domains except the Physical domain, in which alcohol users (median = 6.00) reported significantly higher Physical QOL scores than non-drinkers (median = 5.79) (U = 24,224, *p* = 0.016). There was no significant difference in QOL of life scores between smokers and non-smokers for any of the domains: Emotional (U = 12,302, *p* = .768) Physical (U = 11,100, *p* = .381), Social (U = 12,125, *p* = .785) and Total domains (U = 11,228, *p* = .567).

### Components of QOL at different time periods

The leading components of QOL were compared in the three post-AMI periods (early, 2–10 weeks; intermediate, 5–22 months; and late, > 22 months to 4 years) using linear regression analyses. In the early period, the leading items were “lack of self-confidence” (Emotional domain), “felt excluded” (Physical domain), “felt restricted or limited” (Social domain), and “felt excluded” (Total QOL). In the intermediate period, “lack of self–confidence” for the Emotional domain, and “felt restricted or limited” for the Physical, Social, and total QOL domains, explained the most variance. In the late period (> 22 months to 4 years), “tearfulness”, “unable to socialise”, “felt excluded”, and “unable to socialise” were the leading items for the Emotional, Physical, Social, and total QOL domains, respectively (see Table 2).

**Table 2:** Top QOL items explaining variance in domain scores for patient groups

Patient group	Domain	Top 3 items explaining variance in domain scores	R change (%)	Adjusted R^2^ (%)
2–10 wks post MI	Emotional	15. Lack of self-confidence	70.1	86.1
		10. Tearfulness	11.5	
		2. Worthless or inadequate	5.0	
	Physical	24. Felt excluded	71.9	87.6
		15. Felt restricted or limited	9.6	
		6. Worn out	6.1	
	Social	20. Felt restricted or limited	70.2	90.3
		25. Unable to socialize	14.3	
		15. Lack of self-confidence	6.1	
	Total	12. Felt excluded	67.7	87.5
		15. Lacking self-confidence	15.4	
		10. Tearfulness	4.4	
5–22 months post MI	Emotional	15. Lack of self-confidence	64.7	85.7
		4. Discouraged	15.7	
		8. Restless	5.3	
	Physical	20. Felt restricted or limited	72.6	87.7
		24. Felt excluded	9.4	
		27. Interference with sexual intercourse	5.7	
	Social	20. Felt restricted or limited	70.1	94.7
		12. Unable to do usual social activities	9.9	
		15. Lack of self-confidence	7.5	
	Total	20. Felt restricted or limited	68.4	88.1
		4. Discouraged	15.5	
		12. Unable to do usual social activities	4.4	
> 22 months to 4 yrs. post MI	Emotional	10. Tearfulness	71.1	89.7
		7. Happy with personal life	13.9	
		12. Unable to do usual social activities	4.7	
	Physical	25. Unable to socialize	74.1	90.8
		21. Unsure about exercise	1.05	
		27. Interference with sexual intercourse	6.3	
	Social	24. Felt excluded	89.0	96.4
		21. Unsure about exercise	5.4	
		15. Lacking self-confidence	2.0	
	Total	25. Unable to socialize	77.9	91.7
		18. Apprehensive or frightened	10.9	
		9. Short of breath	2.9	

## Discussion

QOL was found to improve with time following AMI. It was lowest in the first few weeks following AMI, but showed gradual improvement in all QOL domains (physical, emotional, and social) over time with males enjoying better QOL. This finding is consistent with other studies which have demonstrated improvement as time progresses in QOL post AMI [33]. Patients who suffer from NSTEMI, diabetes mellitus, hypertension, and kidney disease (physical and social domain), and IHD (physical domain) had worse QOL. Lifestyle factors such as eating out (once a week), exercising, and alcohol usage were associated with better QOL. Specific QOL item components that accounted for the greatest variance in the early post-AMI period in QOL were lack of confidence, social exclusion, as well as physical restriction or limitation to perform physical activities.

Improved QOL after 1 year compared to 1 week and 5 months was reported by Brink et al. [36]. The lower QOL experienced in the earlier periods post AMI may result from the immediate shock of patients being diagnosed with a heart attack which to many spells doom, despair, lack of energy [37], apprehension [38], shame [39], guilt [39], depression [40], anxiety [40], job loss [41] [42], loss of income [43], and loss or curtailment of social and family life [44]. Interventions to improve QOL in the immediate post-AMI period are therefore critical since psychological and physical counselling and rehabilitation improve clinical outcomes, physical and otherwise [45].

This study identified lack of confidence, tearfulness, social exclusion, inability to socialise, as well as physical restriction or limitation to perform physical activities as some of the main individual items impacting QOL. These variables have also been identified by De Gucht et al. [46]. These may have come about from the efforts of patients to readjust and rehabilitate themselves in the ensuing period of recovery over time [34] [47] [48].

### Age, ethnicity, gender, and QOL

Male patients experienced better QOL as was also found by Dueñas et al. [49]. This may be attributed to males receiving better social support than females as was also reported by Emery et al. [50]. Our study revealed no significant ethnic differences. This contrasts with the reports of Riegel et al. who reported QOL differences among ethnicities (Hispanics, blacks, and whites) [51]. The authors attribute these differences to cultural differences in interpretation of the QOL items. In this study, it is suggested that both the interpretation and experience of QOL are similar across the Indo-Trinidadian, Afro-Trinidadian, and mixed ethnicities.

### Clinical factors and QOL

Significant differences existed between patients with STEMI, diabetes mellitus, hypertension, kidney disease, and IHD, but not hypercholesterolemia. Patients with STEMI had significantly higher median QOL scores than patients with NSTEMI. It has been noted that the size of myocardial damage is related to QOL [52]. This, however was not reflected in this study, in which patients with STEMI and more muscle damage experienced a better QOL (higher median QOL scores) than patients with NSTEMI, as was also reported by Shah et al. [53]. Patients with NSTEMI may be more symptomatic and experience more angina pectoris. Improved QOL is possible when appropriate surgical interventions, drug interventions, counselling, etc. are instituted [54]. There were mixed results with respect to common chronic diseases in this study. In the Physical, Social, and Emotional domains, patients with diabetes had significantly lower QOL scores than patients without diabetes, a finding that was corroborated by Simpson and Pilote [1]. Significant group differences were observed in the Physical and Social domains among patients with kidney disease and hypertension. These contrast with the findings of Wehner and Nitardy, who found that chronic kidney disease did not adversely affect the patient’s QOL 1 year post AMI [55], but is corroborated by Amin et al. who found worsening renal function was associated with reduced QOL [56]. IHD showed lower QOL in the physical domain only, while no significant differences were observed on any QOL domain for patients with hypercholesterolemia. QOL did not vary with hypercholesterolemia, which departs from other study findings [57]. Individuals with hypertension and co-existing co-morbidities tend to have lower QOL than those suffering from hypertension alone [58].

### Lifestyle factors and QOL

Eating out once a week or more was significantly associated with higher QOL in all domains when compared to persons who did not eat out as frequently. Numerous studies have reported dysfunctional eating was associated with poor QOL either directly or indirectly; it is associated frequently with significantly lower QOL scores in the Emotional and Social domains [59]. This study, however, did not measure the quality or type of food or the specific frequency of eating out. In addition, eating out may be related to cultural and personal practices [60] and socializing, which can confer QOL benefits and thus account for the finding. Self-reports of having a stressful life were significantly related to lower median scores in all domains of QOL for all groups. Stress was found to be directly associated with CAD [61] (INTERHEART study). These findings are consistent with those reported by Tasić et al. who found lower QOL in CAD patients with self-reported stress [62]. QOL scores were similar across the Emotional and Social domains as well as total scores for drinkers and non-drinkers. However, alcohol users reported significantly higher physical QOL scores than non-users. This contrasts with Saatcioglu who found alcohol use was related to poorer QOL among patients with alcohol dependence [63]. While alcohol can be used in times of difficulty, it is also associated with festive occasions and may provide culturally accepted opportunities for socializing [64]. Additionally, the patients in this study did not measure the amount or level of alcohol that they consumed; therefore, low or moderate levels of alcohol use may account for the higher QOL scores. It is also noted that improved QOL was only observed in the physical and not social or emotional domains, and as such, the benefits of alcohol use are restricted. From our study, exercising 20 min or more per day was associated with higher QOL, which is consistent with reported literature [65] [66]. No significant group differences in QOL were found between smokers and non-smokers, as was found in another study [67].

#### Limitations

This is a single centre study; however, the findings are still relevant to a national setting. The ratio of male to female patients was approximately 1:1 in the early post-MI period, while in the other two groups, it was at least 2:1. The study used a cross-sectional design with a different cohort of patients at each time point and thus does not reflect changing QOL of the same patients. The broad range of the time periods may also fail to capture potential changes in QOL that occur during these periods. However, even with different cohorts, as used by other studies, QOL trends can be identified. Combining variables for analysis and consideration of multiple comorbidities, although desirable, was deemed inappropriate due to small sample size.

## Conclusion

Increased post-AMI survival, STEMI, non-diabetic, non-hypertensive, non-kidney disease, no self-reported stress, eating out, and exercise were associated with better QOL in all domains. Ethnicity and hypercholesterolemia were not related to QOL. Alcohol was associated with better Physical QOL but not in the Emotional or Social domains. Better QOL was experienced among males and in the younger age groups. Most QOL variance resulted from lack of self-confidence (early and intermediate post AMI), social exclusion (early and late post AMI), restricted feelings (intermediate post AMI), and inability to socialize and tearfulness (late post AMI).

Cardiac rehabilitation should be tailored to meet the needs of different subgroups of cardiac patients based on gender, ethnicity, level of socialization, and level of physical activity. Counselling for issues of self-confidence, social exclusion, and interaction need to be initiated during in-patient stays and continued following hospital discharge. While QOL, a valued outcome, improves over time, cardiac rehabilitation may set the stage for earlier and improved QOL among survivors, particularly among vulnerable groups.

## Abbreviations

AMI: Acute myocardial infarction
ECG: Electrocardiogram
IHD: Ischaemic heart disease
MIST: Myocardial Infarction in South Trinidad
NSTEMI: Non-ST-elevation myocardial infarction
QLMI: Quality of life myocardial infarction
QOL: Quality of life
STEMI: ST-elevation myocardial infarction
WHO: World Health Organization
